# Minimally Invasive Monitoring of Chronic Central Venous Catheter Patency in Mice Using Digital Subtraction Angiography (DSA)

**DOI:** 10.1371/journal.pone.0130661

**Published:** 2015-06-22

**Authors:** Giovanna Figueiredo, Teresa Fiebig, Stefanie Kirschner, Omid Nikoubashman, Lisa Kabelitz, Ahmed Othman, Andrea Nonn, Martin Kramer, Marc A. Brockmann

**Affiliations:** 1 Department of Diagnostic and Interventional Neuroradiology, University Hospital of the RWTH Aachen, Aachen, Germany; 2 Department of Neuroradiology, University Medical Center Mannheim, Medical Faculty Mannheim, Heidelberg University, Mannheim, Germany; 3 Department of Veterinary Clinical Sciences, Small Animal Clinic, Justus-Liebig-University, Giessen, Germany; University of Palermo, ITALY

## Abstract

**Background:**

Repetitive administration of medication or contrast agents is frequently performed in mice. The introduction of vascular access mini-ports (VAMP) for mice allows long-term vascular catheterization, hereby eliminating the need for repeated vessel puncture. With catheter occlusion being the most commonly reported complication of chronic jugular vein catheterization, we tested whether digital subtraction angiography (DSA) can be utilized to evaluate VAMP patency in mice.

**Methods:**

Twenty-three mice underwent catheterization of the jugular vein and subcutaneous implantation of a VAMP. The VAMP was flushed every second day with 50 μL of heparinized saline solution (25 IU/ml). DSA was performed during injection of 100 μL of an iodine based contrast agent using an industrial X-ray inspection system intraoperatively, as well as 7±2 and 14±2 days post implantation.

**Results:**

DSA allowed localization of catheter tip position, to rule out dislocation, kinking or occlusion of a microcatheter, and to evaluate parent vessel patency. In addition, we observed different ante- and retrograde collateral flow patterns in case of jugular vein occlusion. More exactly, 30% of animals showed parent vessel occlusion after 7±2 days in our setting. At this time point, nevertheless, all VAMPs verified intravascular contrast administration. After 14±2 days, intravascular contrast injection was verified in 70% of the implanted VAMPs, whereas at this point of time 5 animals had died or were sacrificed and in 2 mice parent vessel occlusion hampered intravascular contrast injection. Notably, no occlusion of the catheter itself was observed.

**Conclusion:**

From our observations we conclude DSA to be a fast and valuable minimally invasive tool for investigation of catheter and parent vessel patency and for anatomical studies of collateral blood flow in animals as small as mice.

## Introduction

Small rodents such as mice are increasingly used in preclinical research, mostly in the fields of pharmacological, biomedical and behavioral research. As a consequence, repeated vascular access for intravenous or intraarterial administration of medication or contrast agents, as well as for blood sampling may be required. Whereas tail vein injections can be safely performed in mice [1], they sometimes are cumbersome, time-consuming and require training. If performed repeatedly, they may cause venous thrombosis and wounds of the tail interfering with further injections [2].

Using a port-catheter system for long-term vascular catheterization eliminates the need for repeated vessel puncture [3–5]. Pros and cons of different mouse port systems have been described [6]. The most commonly used site for placement of chronic venous catheters in small animals is the external jugular vein [7–10], for which catheter patency beyond 30 days has been reported [11]. Less frequently catheters have also been placed into the femoral vein or the inferior vena cava [7, 12–14]. Whereas long-term catheter placement has been described for pharmacological [15, 16], oncological [17] and cerebrovascular research [4], catheter occlusion is a critical point that may complicate, falsify or even invalidate the results of the studies. Thus, several factors including site of catheter placement, technique of catheter insertion, biocompatibility of the catheter material, diameter and shape of the catheter as well as the schedule for catheter flushing and the type of lock solution used have been discussed to influence catheter patency [11]. Reviewing the literature we found that thrombotic occlusion has been reported to be the most frequent complication of chronic catheterization [2, 18]. This has been reported to be caused by stagnation of blood flow around and within the catheter causing problems like blood clotting, infection or even vessel necrosis [19]. Moreover, “fibrin sleeves” have been described to develop around the intravascular part of the catheter [20–22].

Due to these problems methods to ensure, to extend and to evaluate catheter patency have been discussed. These include withdrawal of blood or injection of saline or other marker-substances through the catheter to evaluate “patency” or “non-patency” as outcome measure [23–26]. During our recent experiments with a novel MRI-compatible micro-port for small animals [6] we were able to gain further insights regarding vessel patency by performing digital subtraction angiography (DSA) in mice [27, 28]. In the underlying manuscript DSA is presented as a method for evaluation of catheter patency.

## Material and Methods

### Animals

All procedures and housing of the animals were carried out according to the criteria outlined in the “Guide for the Care and Use of Laboratory Animals” prepared by the National Academy of Sciences and published by the National Institutes of Health. All experiments were carried out after receiving local ethics committee approval (Regierungspräsidium Karlsruhe). Institutional guidelines for animal welfare and experimental conduct were followed. In the experiments, 10-week-old male C57BL/6J mice, weighing 21–25 g, were used. All animals were kept in a temperature-controlled room with a 12-hour light/dark cycle and had free access to water and a standard laboratory diet.

### Implantation of a vascular access mini-port (VAMP)

Twenty-three mice were anesthetized by an intraperitoneal injection of ketamine (120 mg/kg body weight, Ketamin 10%; Intervet, Bela-Pharm GmbH&CoKG, Vechta, Germany) and xylazine (16 mg/kg body weight, Rompun 2%; Bayer Vital GmbH, Leverkusen, Germany). When sufficient anesthesia was achieved, the animals were positioned supine on a warming plate (Medax GmbH; Neumünster, Germany) at 37°C and fixed with tape. Catheterization of the right jugular vein as well as assembly and implantation of the vascular access mini-port (VAMP) were performed according to procedures previously described [6, 15, 16, 29]. The VAMP was made from a shortened intravenous cannula cone with a rubber membrane inside and with a 5–6 cm long 2F silicone tube (Silastic; Dow Corning, Wiesbaden, Germany), which was slid over the stub of the shortened, stabilized 26 G catheter from the cannula cone. After sterilization at 121°C for 20 min and flushing with heparinized saline solution, we aimed at implanting the catheter tip into the right atrium via the jugular vein. The port reservoir was placed subcutaneously on the animals back.

Postoperatively animals were administered metamizol (Novalgin; Ratiopharm, Ulm, Germany) subcutaneously (200 mg/kg) on the day of the operation and over 3 days via drinking water (200 mg/kg).

The VAMP was flushed every second day with 50 μl of heparinized (25 IU/ml) saline solution. Before flushing, the hair on the back of the animals was clipped and the skin sanitized (Softasept; Braun, Melsungen, Germany) before puncture.

### Digital Subtraction Angiography (DSA)

The animals underwent DSA using the VAMP for contrast injection directly after implantation, as well as one and two weeks after mini-port catheter implantation. DSA was performed using an industrial micro-CT (Y.Fox; Yxlon International GmbH, Hamburg, Germany) with a transmission X-ray tube (Yxlon) and a flat panel detector (Varian PaxScan 2520; Varian, Palo Alto, CA, USA) [27, 28]. Anaesthetized (Forene; Abbot, Wiesbaden, Germany) mice were positioned in a cradle under the X-ray source, gain and offset calibration were performed. DSA was performed by infusion of a pre-warmed (37°C) contrast agent bolus (Iomeprol 300; target volume 100 μl, infusion rate 3.4 ml/min) via the VAMP. DSA projections were acquired at 30 frames per second, using a detector pixel matrix of 944×704, a tube voltage of 80 kV, and a 75 μA current. Spatial resolution of DSA ranged between 18×18 μm and 21×21 μm.

DSA sequences were recorded digitally in an Audio Video Interleave (AVI) movie file format. The AVI files were imported using ImageJ (v1.44i; Wayne Rasband; National Institutes of Health, USA) and decomposed to single projections (by saving them as an image sequence). The decomposed single projections were saved using the RAW file format.

## Results

DSA with administration of 100 μl of an iodine-based contrast agent was technically feasible and tolerated well by all animals. The implanted VAMP and catheter were clearly identifiable in all subtraction angiographies.

**Fig 1:**
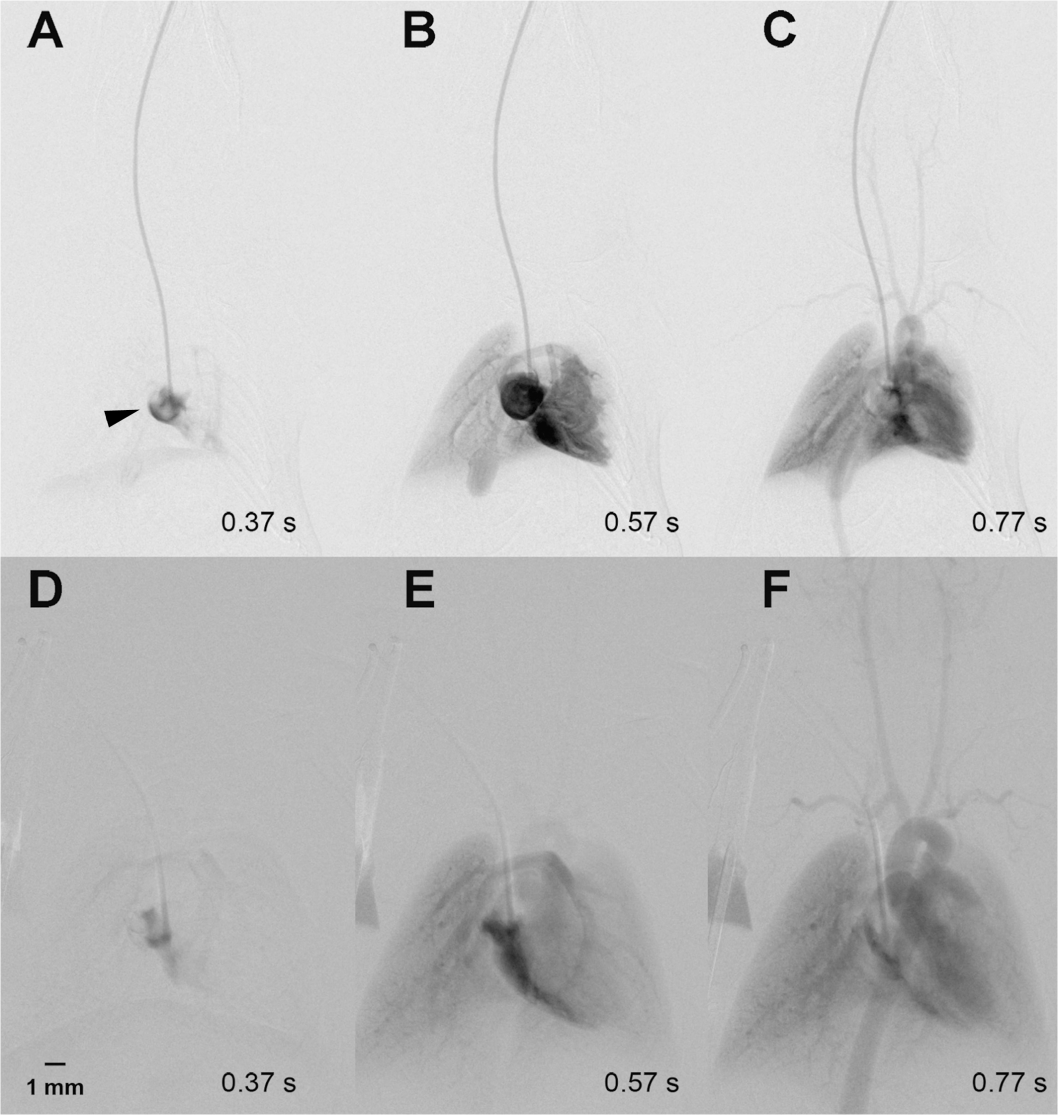
Regular antegrade blood flow pattern. A-C show a regular contrast agent inflow during implantation of a port-catheter into the right atrium (arrow tip) via the right jugular vein. Thus, in A only the right atrium contains contrast agent, which then flows via the pulmonary arteries into the lung (B), and finally contrasts the left ventricle, the aortic arch as well as the supraaortic arterial vessels (C). A repeat DSA with regular contrast agent flow was performed in the same animal 2 weeks later (D-F).

**Fig 2:**
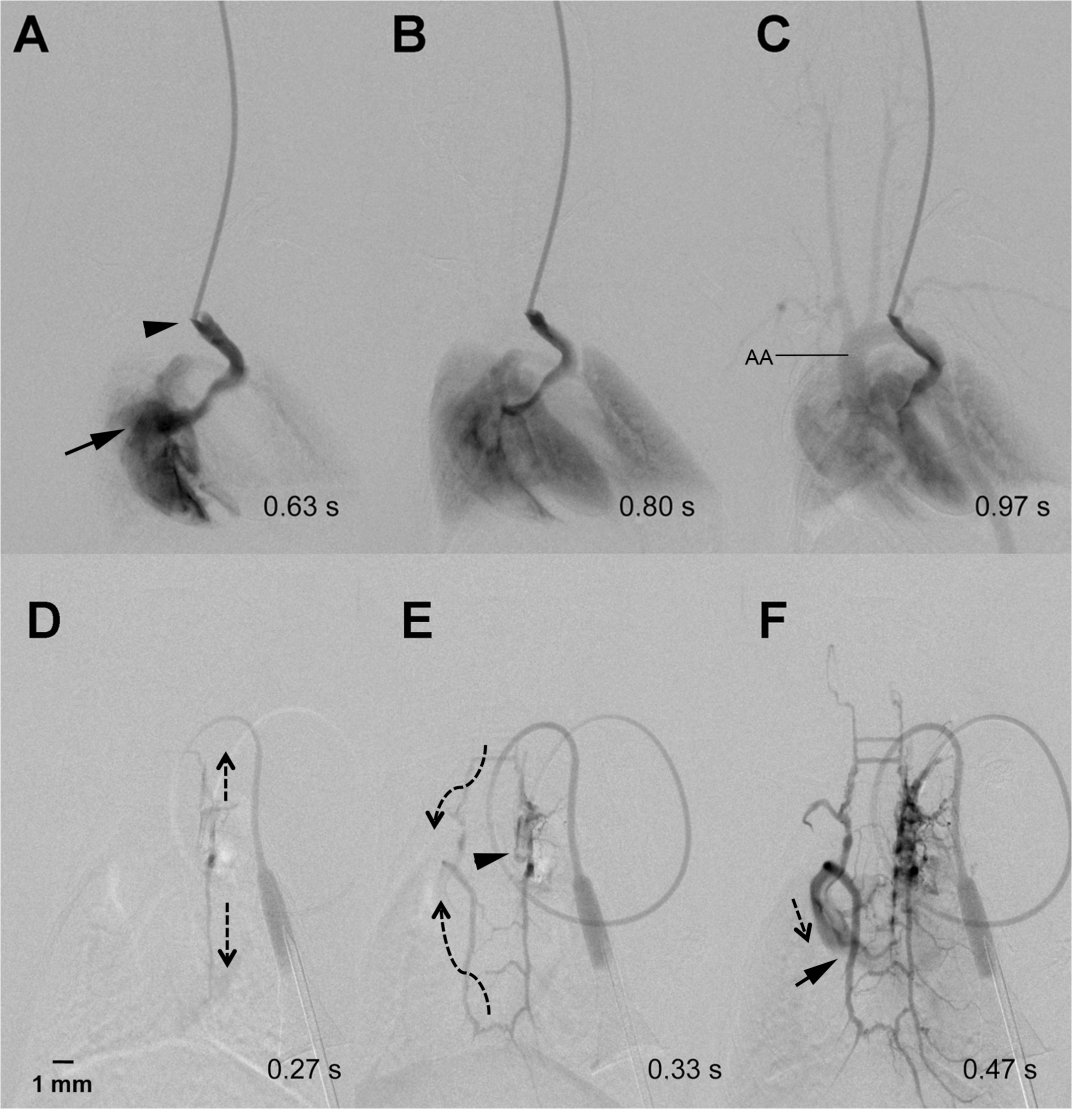
Collateral antegrade flow pattern. The catheter tip (arrow head) is located within the jugular vein of the mouse. Contrast agent flows from the patent jugular vein into the right atrium (arrow) (A). From there it fills the lungs (B) and the aortic arch (AA) including the supraaortic arterial vessels (C). Contrast injection via the implanted mini-port one week later shows occlusion of the jugular vein (D). Instead, the contrast agent flowed (indicated by dotted arrows) via a “ladder-like” venous collateral network (E) with some delay and reduced contrast intensity into the right atrium (arrow) (F).

**Fig 3:**
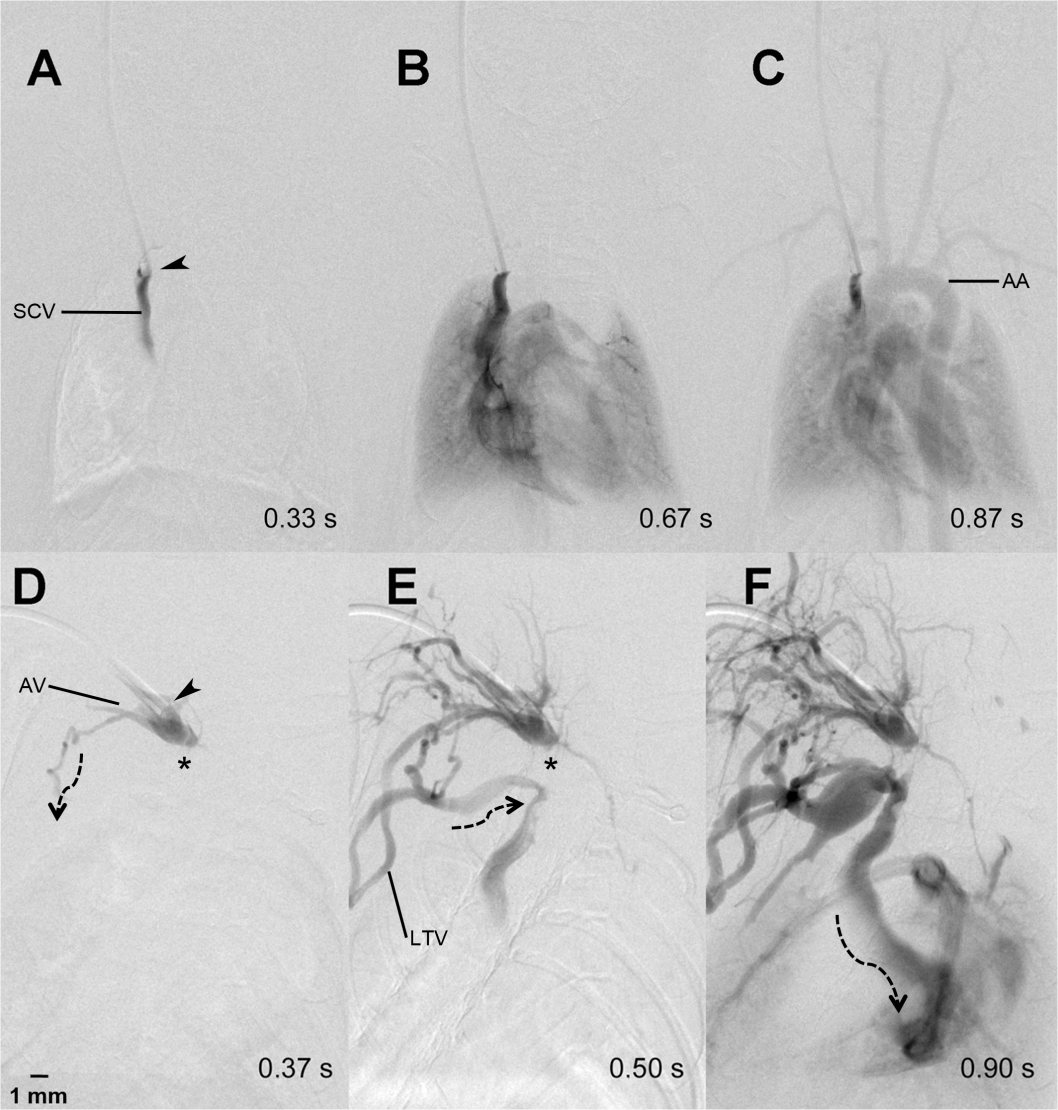
Collateral retrograde flow pattern. Regular contrast agent flow through the heart, lung and aortic arch (AA) after contrast injection into a port-catheter implanted into the superior caval vein (SCV) (A-C). One week later, the catheter tip (arrow head) was dislocated slightly backwards into the confluens of the jugular and axillary vein (AV) and the SCV was thrombosed (*) (D). The contrast agent flowed (dotted arrows) into the proximal SCV via thoracic collaterals including the lateral thoracic vein (LTV) (E and F).

Angiography was carried out directly after the operation in 23 mice. The catheter tip was localized within the right ventricle (n = 1), right atrium (n = 3), superior cava vein (n = 8) and jugular vein (n = 11). In the course of the study, we observed dislocation of three catheters: two dislocated from the superior cava vein into the right atrium and one, positioned in the superior vena cava, dislocated into the proximal jugular vein. Catheter breakage was ruled out in all cases. DSA directly after implantation of the VAMP showed a regular antegrade blood flow pattern with high contrast of the aorta and supra-aortic vessels as shown exemplarily in Fig 1 (and Figs 2A–2C and 3A–3C).

**Fig 4:**
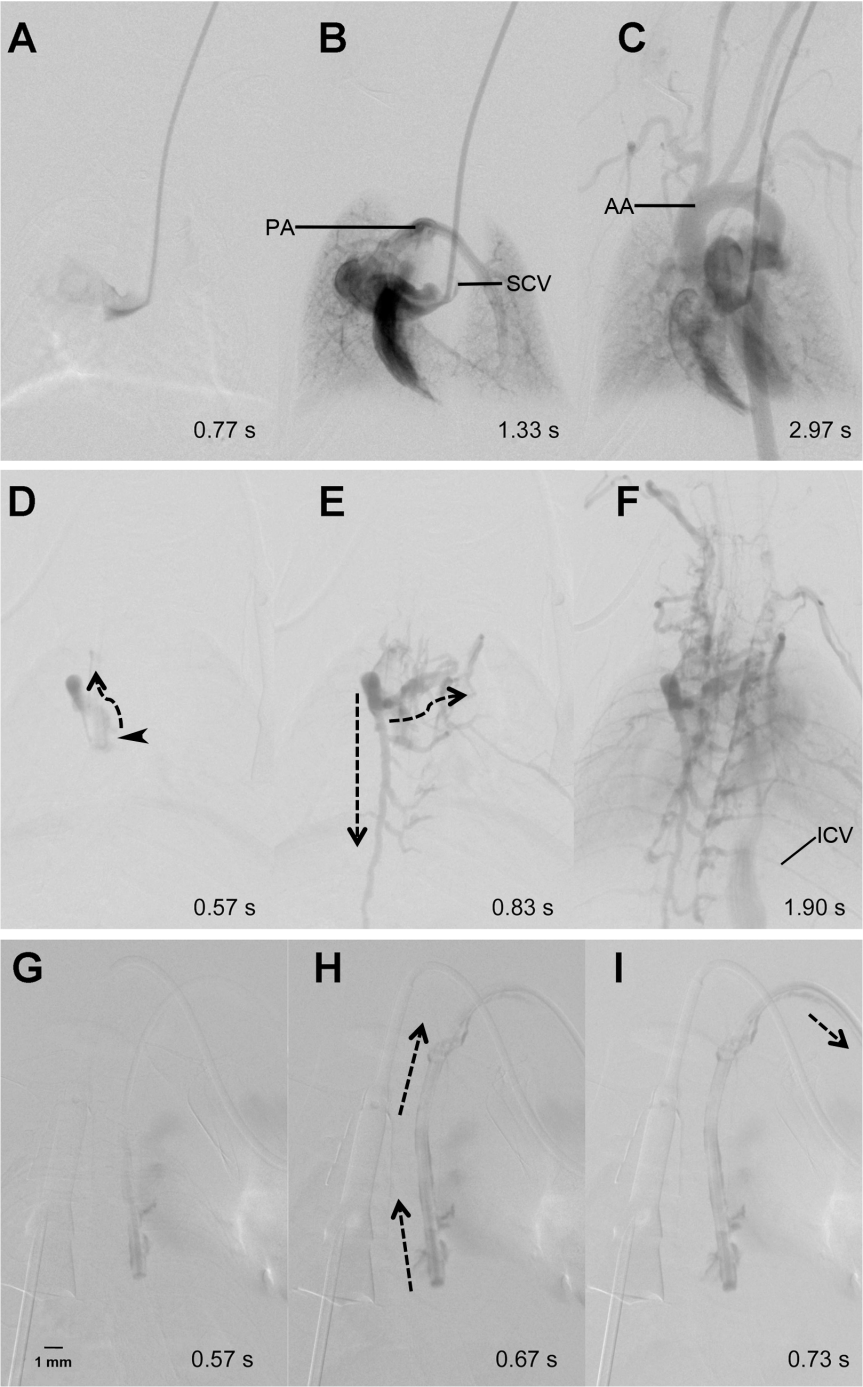
Collateral ante- and retrograde flow pattern. DSA directly after implantation of a catheter port shows timely filling of the superior cava vein (SCV) (A), the right atrium, ventricle, pulmonary arteries (PA), (B) as well as the aortic arch (AA) and supraaortic arterial vessels (C). One week later, the SCV was occluded at the site of the catheter tip (arrow head) (D) and retrograde flow (dotted arrows) of contrast agent leads to filling of perivertebral “ladder-like” and cervicothoracic collateral veins (E). Finally, the intercostal veins (ICV) are contrasted and a faint filling of the right ventricle and the lungs is observed. Another week later (G-I) complete occlusion of the parent vessel is evident. This results in retrograde flow of contrast agent along the outside of the catheter (H and I), which continues outside the jugular vein.

**Fig 5:**
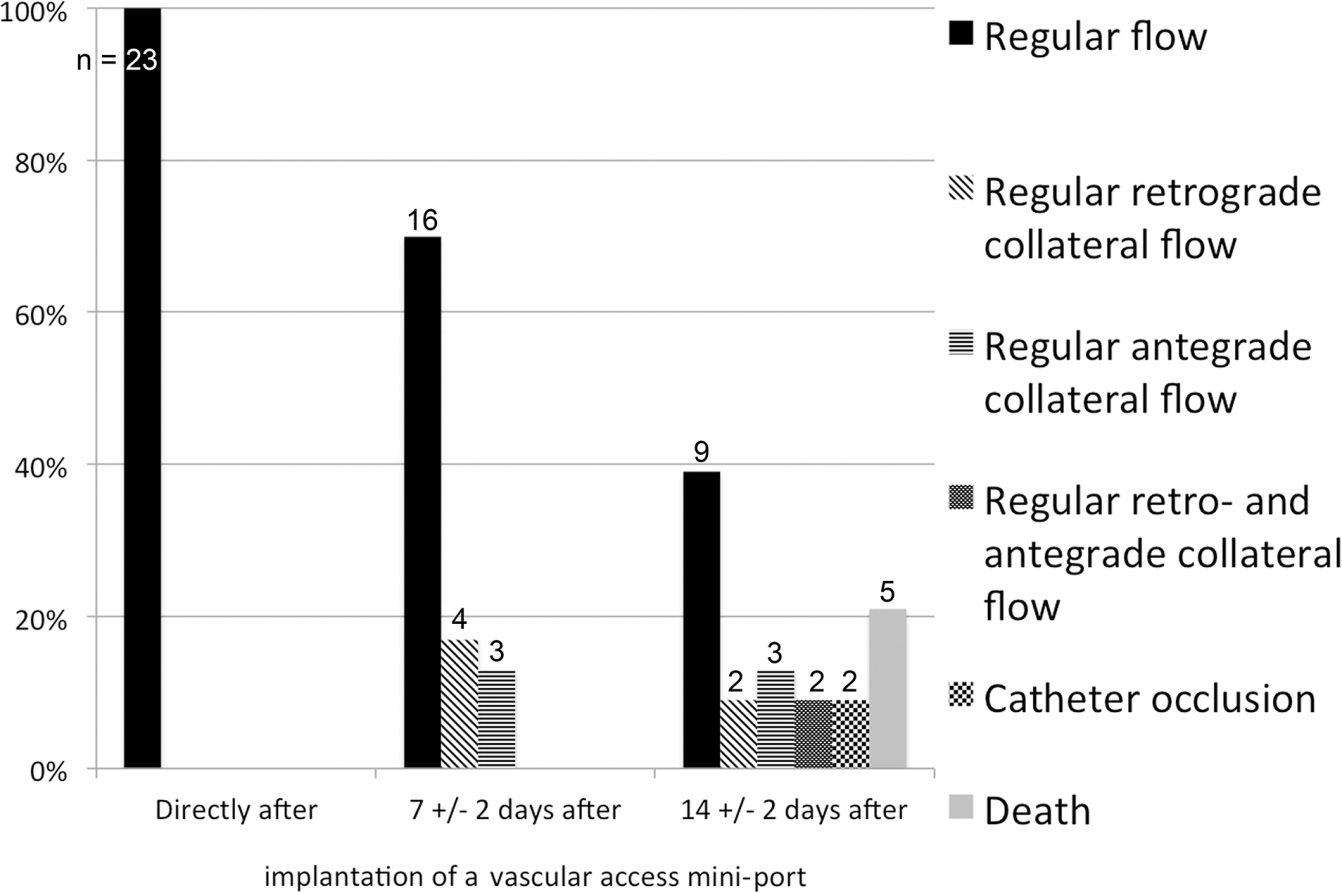
Parent vessel patency and incidence of different flow patterns over the course of two weeks after mini port implantation in mice.

The number of catheters showing a “regular” antegrade blood flow declined over the following two weeks. More exactly, after 7±2 days only 70% (n = 16) of the catheters provided a regular antegrade flow with sufficient arterial contrast. The other 30% (n = 7) presented with different collateral blood flow patterns, which resulted in a reduced arterial contrast in DSA. In all animals showing a collateral flow pattern, however, catheter patency with regard to intravascular administration of contrast agent was confirmed after one week. The following collateral flow patterns were observed: 1.) Occlusion of the jugular vein resulted in contrast enhancement via a “ladder-like” network of perivertebral veins, from which the contralateral jugular vein and the vena cava were contrasted (example shown in Fig 2D–2F). We termed this flow pattern antegrade collateral flow, as the flow filled the collaterals in an antegrade direction. 2.) Occlusion of the jugular vein resulted in blood flow via ipsilateral cervicothoracic collaterals into the vena cava (Fig 3D–3F). This flow pattern we termed “retrograde collateral flow”, as the blood flow filled collaterals in a retrograde direction. 3.) In some cases we visualized a combination of both flow patterns, which we thus termed antegrade and retrograde collateral flow (exemplarily shown in Fig 4D–4F). The incidence of catheter patency and the observed respective flow patterns over the course of two weeks is summarized in Fig 5.

After 14±2 days, 39% (n = 9) of the implanted catheters still provided regular antegrade flow, whereas in 30% (n = 7) of the mice a collateral flow pattern with insufficient arterial contrast was observed. Thus, intravascular application of contrast agent was feasible in 69% of the animals after two weeks. At this time point, 22% (n = 5) of the animals had died or showed complete vessel occlusion (9%; n = 2), as exemplarily shown in Fig 4G–4I. Notably, Fig 4 does not show occlusion of the catheter itself, but only occlusion of the jugular vein up the point of catheter insertion, which results in backflow of contrast agent along the outer side of the catheter even outside the vessel. Cause of death was not identifiable in all animals. Only in 4 animals a (histo-)pathological workup was performed. In one of these animals an intraventricular purulent thrombus was identified, whereas in the remaining animals the cause of death remained unclear.

## Discussion

Regardless of various approaches to maintain catheter patency in small rodents, no studies systematically evaluated the fate of catheters implanted using DSA. Doing so, we found DSA to be a fast and minimally invasive tool for evaluation of parent vessel and catheter patency in live mice. DSA allowed us to intra-operatively verify the catheter position, and helped us to identify catheter dislocation or kinking. Additionally, the high temporal resolution of DSA allowed visualization of collateral blood flow in these small animals.

Whereas catheter occlusion is a frequently discussed topic, we did not observe occlusion of the catheter itself in any of the animals. In all cases, contrast agent injection out of the catheter tip was possible, even though the catheter tip in some cases was located within an occluded parent vessel. In some of these cases, contrast injection still allowed to achieve intravascular enhancement via retrograde flow along the outside of a catheter (within the occluded parent vessel) with subsequent contrast enhancement of collateral vessels (exemplarily shown in Figs 2D–2F and 3D–3F). From this we concluded, that it may still be feasible to use mini-port systems even in case of parent vessel occlusion.

On the other hand, blood flow via collateral vessels leads to a delay and reduction of arterial contrast enhancement, which may be required for radiological high-contrast bolus imaging studies like arterial perfusion imaging. Nevertheless, a port may still be used if a slight delay in contrast enhancement is not of relevance. This, for example, may be the case in studies aiming at medication effects or the visualization of brain tumors in mice [30]. However, one cannot definitely be certain that all quantity of injected medicine will reach the vascular system.

In other cases we observed that the administered contrast agent failed to re-enter the vascular system. In all of these cases, however, DSA provided proof that not catheter, but vessel occlusion was the underlying problem.

In the current experiments we did not try to draw blood via the VAMP. Other authors described the repetitive use of implanted ports or catheters for this purpose in mice. In such a setting, the inability to draw blood from a catheter or port was considered catheter occlusion [16]. Our results indicate, that the inability to draw blood from a venous catheter should not be equated with catheter occlusion or the inability to use a catheter for injections.

Regardless of the mouse port system used, catheter or parent vessel occlusion remains a frequently reported problem. Thus, different factors influencing catheter patency like catheter material and coating [29], the frequency of catheter flushings [4, 29, 31–33], the lock solution used after flushing [33–35], catheter coating [36, 37], as well as form and position of the catheter tip [4, 9, 15, 16, 26, 29, 38, 39] have been discussed elsewhere.

To conclude, DSA allows repeated, fast and definite testing of a port’s patency as well as examination of parent vessel status in animals as small as mice. Our study further demonstrated, that missing antegrade flow during injection does not necessarily prove occlusion of a catheter or port, but that a port still may be used for infusion of medications or contrast agent that do not rely on a bolus technique.
